# Postoperative Prolonged Mechanical Ventilation in Patients With Newly Diagnosed Glioblastoma—An Unrecognized Prognostic Factor

**DOI:** 10.3389/fonc.2020.607557

**Published:** 2020-12-18

**Authors:** Patrick Schuss, Felix Lehmann, Niklas Schäfer, Christian Bode, Elisa Scharnböck, Christina Schaub, Muriel Heimann, Anna-Laura Potthoff, Johannes Weller, Erdem Güresir, Christian Putensen, Hartmut Vatter, Ulrich Herrlinger, Matthias Schneider

**Affiliations:** ^1^Department of Neurosurgery, University Hospital Bonn, Bonn, Germany; ^2^Department of Anesthesiology and Critical Care Medicine, University Hospital Bonn, Bonn, Germany; ^3^Division of Clinical Neuro-Oncology, Department of Neurology, University Hospital Bonn, Bonn, Germany

**Keywords:** prolonged mechanical ventilation, glioblastoma, overall survival, cancer, brain tumor

## Abstract

**Objective:**

Although the treatment of glioblastoma patients is well established in neuro-oncological surgery, precious scarce data is available on patients with glioblastoma requiring postoperative prolonged mechanical ventilation (PMV). Therefore, the aim of the present study was to determine the influence of PMV on overall survival (OS) in patients with glioblastoma.

**Methods:**

Patients with newly diagnosed glioblastoma who had undergone surgical therapy and complete subsequent neuro-oncological treatment at the authors’ neuro-oncological center from January 2013 to December 2018 were selected and included in the further analysis. PMV was defined as mechanical ventilation for more than 24 h after surgery. Survival analyses were performed, including established prognostic factors such as age, Karnofsky performance score, MGMT-promoter methylation status and extent of resection.

**Results:**

A total of 240 patients with newly diagnosed glioblastoma and subsequent surgical treatment were identified. 13 patients (5%) suffered from PMV during the treatment course of glioblastoma. All but one patient were successfully weaned from mechanical ventilation. Patients suffering from PMV achieved significantly less often favorable functional outcome after 3, 6, 9, and 12 months compared to patients without PMV. Multivariate analysis revealed PMV to constitute a significant prognostic factor for OS, independent of other prognostic factors (p<0.0001, OR 6.7, 95% CI 3.2–13.8).

**Conclusions:**

The present study identifies PMV as significantly associated with impaired functional outcome and poor OS in patients suffering from newly diagnosed glioblastoma. These findings encourage further efforts to investigate/assess this prognostic factor in future studies.

## Introduction

The advancements in modern intensive care medicine have saved numerous lives (1). One of the challenges it entails are patients for whom intensive care enables survival in the acute phase of critical illness, but for whom full recovery is almost impossible because of a failure of mandatory subsequent treatment initiation. Mechanical ventilation is a fundamental component of intensive care medicine, and a continued dependence on mechanical ventilation after an acute episode of ICU care/monitoring is an indication of chronic critical illness (2).

However, prolonged mechanical ventilation (PMV) affects overall survival, especially in cancer patients (3). Due to the long-lasting reduced state of health, patients are often not able to tolerate additional—yet necessary—adjuvant therapy regimens. This leads to a relevant postponement or withdrawal of treatment. Although neurological conditions are considered to be important, little is known about the characteristics that lead to PMV in neurological/neurosurgical patients (4, 5).

Although several studies include the influence of PMV on various diseases of neurosurgical patients, we are not aware of any previous detailed reports analyzing a potential prognostic impact of PMV in glioblastoma disease. Therefore, the present study was aimed at determining the influence of PMV on overall survival (OS) in patients with newly diagnosed glioblastoma.

## Materials and Methods

### Patients

All patients with newly diagnosed glioblastoma who underwent surgical treatment on at the authors’ facility between 2013 and 2018 were entered into a computerized database (SPSS, version 25, IBM Corp., Armonk, NY). Approval for this study was granted by the institutional ethics committee. In order to exclude the surgical treatment as a confounding factor, we excluded patients who underwent biopsy alone instead of surgical resection from further analysis.

Information, including patient characteristics, radiological features, MGMT-promoter methylation status, functional neurological status at admission and during the course of treatment was recorded and further analyzed. The Karnofsky performance score (KPS) was used to evaluate patients according to their neurological functional status preoperatively, postoperatively, and during follow-up (at 3, 6, 9, and 12 months). In this context, KPS ≥ 70 was defined as a favorable outcome. Treatment decisions were made at the initial presentation of the patient and during follow-up by the institutional interdisciplinary tumor advisory board meetings for the Central Nervous System, as described previously (6). Extent of resection (EOR) was assessed in early (<72 h) postoperative magnetic resonance imaging (MRI, 3T). Gross-total resection (GTR) was determined as complete removal of the contrast-enhancing tissue (i.e., absence of residual enhancing tumor tissue).

Patients’ comorbidity burden at admission was assessed according to the Charlson comorbidity index (CCI). Patients were divided into two groups according to their comorbidity burden after corresponding age adjustment: lower comorbidity burden (age-adjusted CCI 0–4) and higher comorbidity burden (age-adjusted CCI ≥5) (7).

PMV was defined as prolonged postoperative invasive ventilation for more than 24 h, as recently indicated (8).

Overall survival (OS) was measured from the day of glioblastoma surgery until death or last observation. All parameters were compared in terms of OS.

### Statistics

Data analysis was performed using the computer software package SPSS (version 25, IBM Corp., Armonk, NY). Unpaired categorical and binary variables were analyzed in contingency tables using the Fisher’s exact test. The Mann-Whitney U-test was chosen to compare continuous variables as the data were mostly not normally distributed. OS was analyzed by the Kaplan-Meier method. The log-rank test was used to compare survival rates by sex, age at diagnosis (≤65, >65 years), preoperative KPS (<70, ≥70), extent of resection (GTR, STR) and PMV (mechanical ventilation ≤24 h, >24 h). Relevant clinical factors were entered into multivariable Cox proportional risk models to predict overall survival. Results with p<0.05 were considered statistically significant.

## Results

### Patient Characteristics

Between 2013 and 2018, a total of 413 patients were treated for newly diagnosed glioblastoma at the authors’ neuro-oncological center. 173 patients were excluded from further analysis after careful review of the clinical records. This was done either because these patients received only biopsy alone and/or because the preoperatively determined therapy framework did not permit a further intensive care treatment according to the patient’s wishes. Therefore, 240 patients with newly diagnosed glioblastoma were included in further analysis. The median age was 64 years (range 19–86 years). At admission, patients presented with a median KPS score of 90. GTR was performed in 164 patients (68%). 13 patients with newly diagnosed glioblastoma suffered from postoperative PMV (5%). Median overall survival for patients with histologically proven newly diagnosed glioblastoma was 16 months (95% CI 14.4–17.6).

### Patients With Glioblastoma and PMV

In total, 13 patients (5%) with newly diagnosed glioblastoma developed PMV during the course of postoperative treatment. Patients who experienced PMV after surgery suffered significantly more often from postoperative complications than patients without PMV (17% vs. 85%; p<0.0001, OR 26.5, 95% CI 5.7–124.4). In detail, patients with PMV developed postoperative pneumonia in a significantly higher proportion than patients without PMV (46% vs. 2%; p<0.0001, OR 38.1, 95% CI 9.3–155.2). However, regarding the comorbidity burden at admission, patients with PMV after surgical glioblastoma resection presented with a significantly higher CCI compared to patients without PMV (46% vs. 16%; p=0.01, OR 4.5, 95% CI 1.4–14.3). Preoperative differences in patient characteristics between patient with and without PMV after glioblastoma surgery are given in detail in **Table 1**.

**Table 1:** Preoperative patient characteristics.

	Patients without PMV (n=227)	Patients with PMV (n=13)	p-value
Median age at operation	63	69	n.s.
Female sex	92 (41%)	4 (31%)	n.s.
Median preoperative KPS	90	80	n.s.
Median BMI	26	26	n.s.
Age-adjusted CCI ≥ 5	36 (16%)	6 (46%)	p=0.01, OR 4.5, 95% CI 1.4–14.3
CCI: COPD	8 (4%)	0 (0%)	n.s.
CCI: liver disease	1 (0.4%)	2 (%)	p=0.008, OR 41.1, 95% CI 3.5–488.5
CCI: diabetes mellitus	24 (11%)	4 (31%)	n.s.
CCI: CKD	6 (%)	0 (0%)	n.s.
Preoperative CRP > 5 mg/l	34 (15%)	1 (8%)	n.s.
Preoperative WBC > 12 G/l	93 (41%)	8 (62%)	n.s.

PMV, prolonged mechanical ventilation; n.s., not significant; KPS, Karnofsky performance score; BMI, body mass index; CCI, Charlson comorbidity index; OR, odds ratio; CI, confidence interval; COPD, chronic obstructive pulmonary disease; CKD, chronic kidney disease; CRP, c-reactive protein; WBC, white blood cells.

Patients with PMV often did not receive adjuvant radio- and/or chemotherapy postoperatively due to their poor neurological/physical condition (54%). In total, four patients with PMV (31%) were discharged postoperatively to their homes following hospitalization. three of the patients with PMV (23%) were referred to a rehabilitation institution in order to achieve a relevant improvement of their poor neurological/physical condition for further adjuvant therapy. Three patients with PMV (23%) were discharged to a nursing home or hospice. Further details on potential influencing factors in patients with PMV are given in **Table 2**.

**Table 2:** Characteristics of patients with PMV and glioblastoma.

Patient No.	Age/sex	Duration of PMV (h)	Age-adjusted CCI	ASA	Postoperative complication	Postoperative status epilepticus	Adjuvant radio- and/or chemotherapy	Postoperative discharge destination
1	82, f	30	6	3	pneumonia, pulmonary embolism	yes	yes	home
2	43, f	121	0	1	SSI	yes	no	nursing home, palliative care
3	67, f	696	8	2	pneumonia	yes	no	rehabilitation institution
4	81, m	78	7	3	myocardial infarction	no	no	rehabilitation institution
5	69, m	104	2	2	no	no	yes	home
6	58, m	34	1	2	epidural hemorrhage	no	yes	home
7	72, m	27	3	2	epidural hemorrhage, SSI	no	yes	home
8	77, f	73	6	3	CSF fistula	no	yes	nursing home
9	76, m	140	5	2	infarction, hemorrhage, pneumonia	no	no	hospice
10	74, m	157	6	3	pneumonia	no	no	in-hospital death
11	21, m	217	0	2	hemorrhage, postoperative seizures	yes	no	rehabilitation institution
12	59, m	312	2	2	pneumonia	no	no	in-hospital death
13	60, m	47	4	3	no	no	yes	home

ASA, American society of anesthesiologists; CCI, Charlson comorbidity index; CSF, cerebrospinal fluid; f, female; m, male; PMV, prolonged mechanical ventilation; SSI, surgical site infection.

### Influence of PMV on Functional Outcome

**Figure 1:**
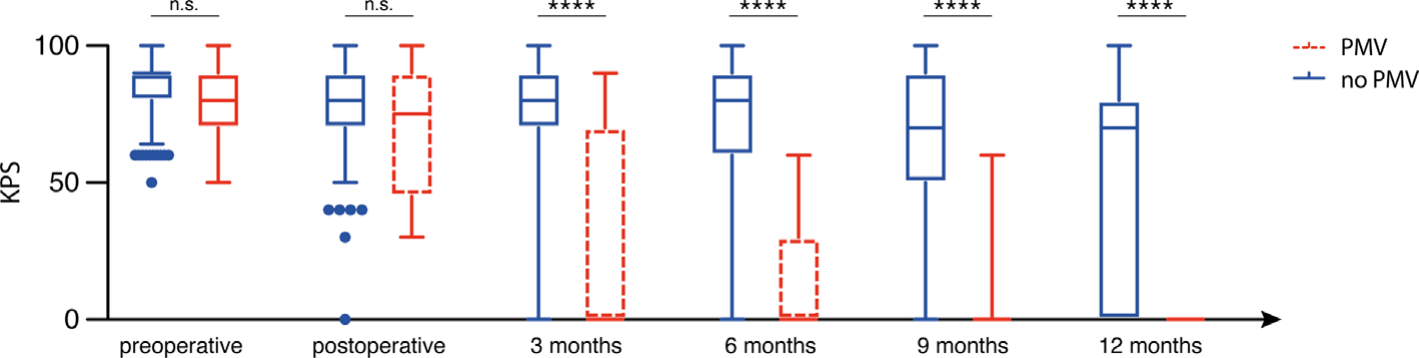
Prolonged mechanical ventilation (PMV) is associated with impaired postoperative functional outcome. n.s., not significant, ****p < 0.001.

Patients without PMV presented with a preoperative median KPS of 90 (95% CI 80–90), whereas patients with PMV presented with a median KPS of 80 (95% CI 70–90; p=0.07). Subsequently, median KPS in patients with PMV did not differ significantly from patients without PMV during the direct postoperative time period (p=0.3). Nevertheless, median KPS in patients with PMV differed significantly compared to patients without KPS at 3 months after surgery (p<0.0001, **Figure 1**).

After dichotomization for KPS, the number of patients without PMV and with a preoperative KPS ≥ 70 did not differ significantly compared to patients with PMV (216/11 vs. 11/2; p=0.2). Immediately after surgery, favorable KPS in patients with PMV did significantly differ compared to patients without PMV (p=0.0003, OR 9.1, 95% CI 2.8–29.4). Moreover, patients with PMV significantly less frequently achieved a favorable KPS ≥ 70 at the 3 months (p=0.0003, OR 8.9, 95% CI 2.6–30.0), 6-months (p<0.0001, OR 29.9, 95% CI 3.8–234.8), 9-months (p<0.0001, OR 24.3, 95% CI 3.1–190.7) and 12-months (p=0.0002, OR 28.2, 95% CI 1.7–480.6) follow-up visits.

### Influence of PMV on Overall Survival/Mortality

**Figure 2:**
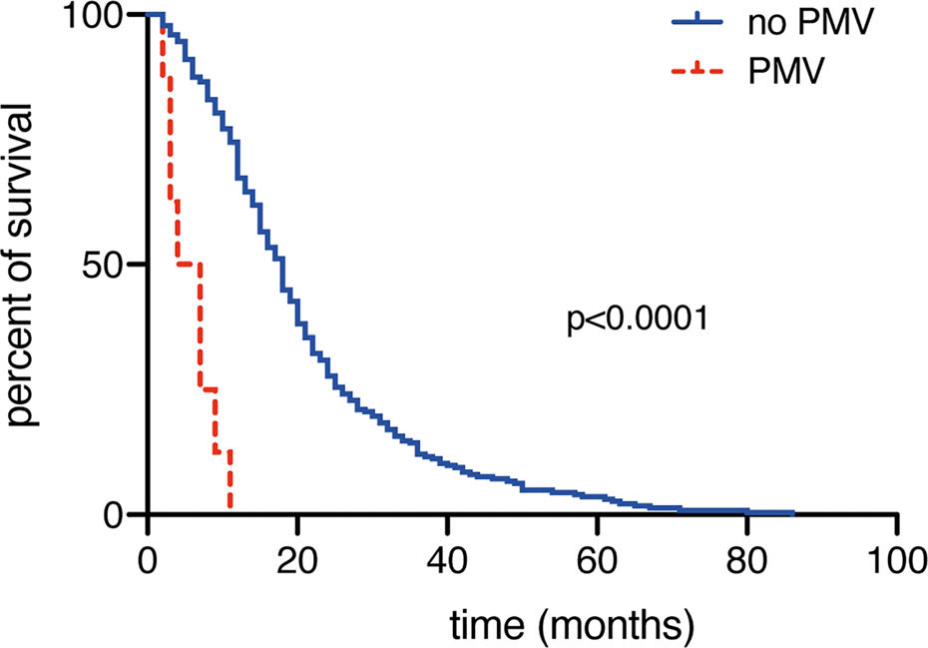
Prolonged mechanical ventilation (PMV) is associated with decreased overall survival rates.

**Figure 3:**
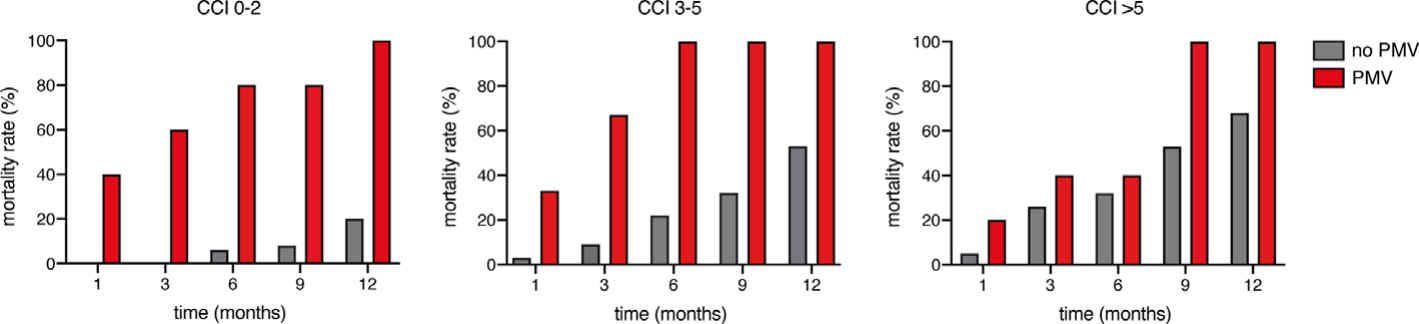
Influence of comorbidities and prolonged mechanical ventilation (PMV) on mortality rates.

Patients with newly diagnosed glioblastoma who suffered from PMV achieved a median OS of 3 months (95% CI 1.2–4.8), whereas patients without PMV achieved an OS of 18 months (95% CI 16.2–19.8; p<0.0001; **Figure 2**). Mortality at 30 days was significantly higher in patients suffering from postoperative PMV compared to patients without PMV (31% vs. 2%; p=0.0003, OR 24.8, 95% CI 5.3–115.4). Patients with postoperative PMV exhibited significantly higher 3 months mortality rates compared to patients without PMV (54% vs. 6%; p<0.0001, OR 19.2, 95% CI 5.6–65.4). Furthermore, mortality after 1 year significantly differed between patients without and with PMV (34% vs. 100%; p=0.0002, OR 33, 95% CI 1.9–579.9). The influence of PMV development and the corresponding comorbidity burden in patients with glioblastoma on mortality rates after 1, 3, 6, 9, and 12 months are shown in **Figure 3**.

### Multivariate Analysis

We conducted an additional multivariate survival analysis to identify independent predictors of OS in patients with glioblastoma. The multivariate analysis identified the known important variables like “subtotal resection” (p=0.01, OR 1.5, 95% CI 1.1–2.0), “unmethylated MGMT promoter status” (p<0.0001, OR 1.9, 95% CI 1.4–2.6), and “age ≥ 65 years” (p<0.0001, OR 2.4, 95% CI 1.8–3.2) as significant and independent predictors for a poor overall survival. Furthermore, the above-mentioned model also identified “PMV” as a significant and independent predictor for poor overall survival in patients with newly diagnosed glioblastoma (p<0.0001, OR 6.7, 95% CI 3.2–13.8).

## Discussion

The present study identifies PMV as a so far unrecognized independent prognostic factor for impaired OS in patients with newly diagnosed glioblastoma.

PMV has been described earlier as an important outcome parameter in patients with various types of cancer (9, 10). However, previous studies show that the influence of the type of underlying malignancy on weaning outcomes was not as relevant as the conditions and parameters associated with the chronic critical illness in terms of respiratory function (11). Shih and colleagues described a 1-year survival rate of 14% in cancer patients requiring PMV (3). The necessity of mechanical ventilation for more than 96 h resulted in a significant impairment of quality of life and functional status, but had no significant impact on survival in previous studies (9, 10). In comparison to the reported findings regarding PMV in other cancer patients, the patient’s co-morbidity burden does not seem to play a significant role concerning the aggravated postoperative weaning situation and subsequent development of PMV in the present study population.

In addition to the aforementioned implications in critically ill cancer patients, patients with cancer of the central nervous system pose a particular challenge. In addition to the equally possible physical predisposition to a postoperative complex respiratory weaning situation, these patients also have the risk of a disease-related impairment of vigilance, which on its own may result in the necessity of ventilation. In the current patient population, 38% of those patients affected by postoperative PMV also exhibited postoperative status epilepticus. Tumor-associated status epilepticus seems to evince a more benign course compared to the non-tumorous status epilepticus, but is associated with a poor prognosis, particularly in patients with aggressive brain tumors (12).

Due to a relevant impairment (postoperative complications, status epilepticus, PMV) of the physical condition of affected glioblastoma patients, a delayed initiation of a necessary adjuvant therapy is often unavoidable. In cases with considerable delay, this leads to a decreased overall survival (13). This is consistent with the finding that the majority of patients with PMV in the present study did not receive adjuvant therapy due to prolonged intensive care and associated poor neurological/physical status. This must be taken into account when assessing the impact of PMV on OS in glioblastoma patients.

In principle, the discussion about the benefit of a prolonged intensive care treatment for glioblastoma patients must also become apparent. Nevertheless, intensive care medicine represents an important element in the continuum of modern cancer therapy (14). After a verified histopathological diagnosis, a re-evaluation of a further therapy desire considering an expected intensive care therapy is feasible under consideration of the patient’s wish. It is also conceivable to attempt an improvement of the neurological/physical condition during a short-time rehabilitation period in order to reach a therapeutical capability.

Nevertheless, the results of the present study are meant to raise awareness of a specific and relatively small patient population with very high mortality and poor functional outcomes, that is those requiring at least 24-h of mechanical ventilation in the postoperative course. This patient group is difficult to identify preoperatively due to the limited data available. However, knowledge of the factors associated with mortality in these patients would be of great clinical interest. In the course of the numerous discussions about the most effective therapy for patients with glioblastoma, optimal counseling/care in the event of a significantly worsening prognosis is also becoming important in the context of patient self-determination. In order to be able to inform, accompany and advise patients/relatives/caregivers well and comprehensively, the assessment of the relevance of clinical setbacks - such as PMV - is essential for clinicians.

### Limitations

The present study has several limitations. The data collection was conducted retrospectively. Patients were not randomized, but treated according to the preferences of the treating physicians. Furthermore, the relevant patient group appeared to be very small and therefore hardly allows any conclusions to be drawn about the underlying causes of PMV. Nevertheless, the present study investigates this aspect for the first time in glioblastoma patients and thus provides the basis for the initiation of multicenter registries and further studies.

## Conclusions

The present study suggests PMV to constitute a novel independent predictor for poor functional outcome and increased mortality following neurosurgical treatment in patients with newly diagnosed glioblastoma. Therefore, considerably more clinical attention ought to be devoted to neuro-oncological patients who require PMV. Further research on this matter is urgently needed in order to enable early preoperative detection of those highly vulnerable glioblastoma patients.

## Data Availability Statement

The original contributions presented in the study are included in the article. Further inquiries can be directed to the corresponding author.

## Ethics Statement

The studies involving human participants were reviewed and approved by Local Ethics Committee at the University Hospital Bonn. Written informed consent for participation was not required for this study in accordance with the national legislation and the institutional requirements.

## Author Contributions

Conceptualization: PS, MS, UH. Methodology: PS, MS, NS, UH. Data collection: PS, FL, NS, CB, ES, CS, MS, A-LP, JW, MS. Statistics: PS, MS, A-LP. Figures: MS, A-LP, PS. Writing—original draft: PS, MS. Study supervision: PS, UH, MS. Proof-reading: all authors. All authors contributed to the article and approved the submitted version.

## Conflict of Interest

The authors declare that the research was conducted in the absence of any commercial or financial relationships that could be construed as a potential conflict of interest.
